# Surgical Approaches and Perioperative Outcomes in Mediastinal Paragangliomas: A 20-Year Comprehensive Systematic Review

**DOI:** 10.3390/cancers18030486

**Published:** 2026-02-01

**Authors:** Nicola Rotolo, Giorgia Cerretani, Sabrina Casagrande, Elisa Nardecchia, Elena Asteggiano, Alberto Colombo, Luca Filipponi, Filippo Piacentino, Schiorlin Ilaria, Federico Fontana

**Affiliations:** 1 Thoracic Surgery Unit, Department of Medicine and Technological Innovation, University of Insubria, Ospedale di Circolo, 21100 Varese, Italy; acolombo1@studenti.uninsubria.it; 2 Thoracic Surgery Unit, Department of Medicine and Surgery, University of Insubria, Ospedale di Circolo, 21100 Varese, Italy; 3 Department of Cardio-Thoraco-Vascular Diseases, Fondazione IRCCS Ca’ Granda-Ospedale Maggiore Policlinico, University of Milan, 20122 Milan, Italy; giorgia.cerretani@unimi.it; 4 Nuclear Medicine Unit, ASST Settelaghi, 21100 Varese, Italy; sabrina.casagrande@asst-settelaghi.it (S.C.); ilaria.schiorlin@asst-settelaghi.it (S.I.); 5 Thoracic Surgery Unit, Ospedale ASST-Settelaghi, 21100 Varese, Italy; elisa.nardecchia@asst-settelaghi.it (E.N.); elena.asteggiano@asst-settelaghi.it (E.A.); luca.filipponi@asst-settelaghi.it (L.F.); 6 Diagnostic and Interventional Radiology Unit, ASST Settelaghi, Insubria University, 21100 Varese, Italy; filippo.piacentino@asst-settelaghi.it (F.P.); federico.fontana@uninsubria.it (F.F.)

**Keywords:** mediastinal paraganglioma, surgical resection, cardiopulmonary by-pass, post-operative complications, systematic review

## Abstract

Background: Mediastinal paragangliomas (MPs) are rare, highly vascular neuroendocrine tumors whose surgical resection is the gold standard but carries a high risk of perioperative complications due to the MPs’ proximity to major cardiovascular structures with potential life-threatening hemorrhage. Due to their rarity, the literature consists primarily of case reports. Our systematic review aims to synthesize the evidence from the last two decades to propose a standardized, multidisciplinary approach to the diagnosis and surgical management of MPs. Methods: A systematic literature review was conducted from 2005 to 2025. Studies reporting on surgically resected adult mediastinal paragangliomas were included. Patient demographic data, diagnostic workup, surgical approaches, and outcomes were extracted and analyzed descriptively. Results: Analysis of 79 patients from 75 papers revealed a median age of 50 years (female predominance of 62%). Most tumors were in the middle mediastinum (51.9%). Sternotomy was the most common surgical approach (44.3%), with cardiopulmonary bypass utilized in 27.8% of cases. Postoperative complications occurred in 28% of patients, with left vocal cord palsy (12.7%) being most frequent. The median follow-up was 12 months. All percentages refer to the number of patients. Conclusions: Surgical removal is the standard of care for curative treatment of MPs. However, surgical treatment requires meticulous planning within a multidisciplinary team to reduce the risk of perioperative complications. The choice of surgical approach—open, minimally invasive, or with circulatory support—depends on tumor site, size, and vascular involvement. This review consolidates existing evidence of MPs’ surgical management, aiming to mitigate the significant risks associated with surgery. Lifelong follow-up is essential due to the potential for recurrence.

## Simple Summary

This study reviews the surgical management of a mediastinal paraganglioma, a rare type of tumor that is often located in the posterior mediastinum and can surround or involve the heart and major blood vessels. Often asymptomatic or with symptoms related to catecholamine secretion, the surgical approach is the treatment of choice, achieving local disease control and long-term outcomes. However, surgical removal poses a high risk of severe bleeding and perioperative complications. By analyzing literature from the last twenty years, we aim to establish a clearer and safer approach for diagnosis and surgery. The findings will help surgeons better plan these complex operations, potentially reducing complications and improving patient care for this uncommon but dangerous condition.

## 1. Introduction

Mediastinal paragangliomas (MPs) are rare neuroendocrine tumors arising from extra-adrenal chromaffin cells, accounting for less than 2% of all paragangliomas and fewer than 0.3% of mediastinal neoplasms [1,2,3,4,5]. They can originate at various anatomical sites and are classified as adrenal or extra-adrenal paragangliomas. MPs represent an exceptionally rare subset, accounting for a very small proportion of extra-adrenal cases. From a biological standpoint, these tumors may be functional or non-functional depending on catecholamine secretion, and their clinical behavior ranges from indolent to locally aggressive, with malignant potential defined by the presence of metastases [6].

Given their rarity, anatomical complexity, and surgical challenges, MPs represent a unique clinical entity for thoracic surgeons. They pose significant challenges in diagnosis and surgical treatment due to their location and potential catecholamine secretion, necessitating advanced imaging modalities, biochemical testing [2,3,4,5,6,7], and careful perioperative management, including appropriate pharmacological preparation and meticulous surgical planning, to minimize complications. Despite the limited availability of prospective studies due to the disease’s rarity, international guidelines recommend surgical resection as the treatment of choice, highlighting the favorable postoperative outcomes, excellent long-term survival, low recurrence rates, and acceptable morbidity [8,9]. However, its position close to the mediastinal vessels, sometimes surrounded or involved by the tumor, makes surgical intervention very dangerous due to the risk of potentially fatal intra-operative hemorrhages. To reduce this risk, the surgical approach must be evaluated with caution [1,2,4,5,7,10,11,12,13].

This paper aims to provide a comprehensive descriptive synthesis of current surgical strategies and perioperative outcomes through a literature review from the past two decades, which largely consists of case reports and case series. Our analysis specifically addresses diagnostic methodologies and surgical interventions, supported by practice-based clinical evidence.

## 2. Materials and Methods

### 2.1. Search Plan

A comprehensive literature search was conducted across three major databases: PubMed, Scopus, and Web of Science. Specific search strings were developed for each database using a combination of free-text terms tailored to the syntax of each platform. The search strategy aimed to identify studies reporting on surgically resected mediastinal or thoracic paragangliomas from 2005 to 2025. The search terms included “paraganglioma,” “extra-adrenal paraganglioma,” “thoracic paraganglioma,” “mediastinal paraganglioma,” “thorax,” “mediastinum,” “surgery,” “surgical resection,” “surgical treatment,” and “surgical complications.” All databases were last searched on 25 August 2025. The full search strategies and exact search strings used for each database are provided in the Supplementary Materials (S1).

Inclusion criteria for studies included mediastinal paragangliomas in adults of both sexes who had undergone surgical resection. Exclusion criteria included pediatric paragangliomas, extra-mediastinal paragangliomas, metastatic lesions, and articles not in English or with inconsistent data. The present review was limited to surgically treated MPs to specifically analyze surgical approaches and perioperative outcomes. Non-surgical management strategies, such as active surveillance or other non-surgical treatment, were consequently outside the scope of this analysis. Retrieved records were organized in a dedicated database for screening and data management in accordance with guidelines from the Preferred Reporting Items for Systematic Reviews and Meta-Analyses statement (PRISMA) [14]. To avoid duplication of patient data, review articles and overview papers were not included in the quantitative synthesis but considered separately and discussed. The systematic review adhered to the PICO (Population, Intervention, Comparison, and Outcomes) framework as follows: Population: patients with mediastinal paraganglioma; Intervention: surgical resection; Comparison: no comparator group; Outcomes: perioperative complications and follow-up [15].

Given the rarity of MPs, most included studies consisted of case reports and small case series. Therefore, a formal quality assessment or risk-of-bias evaluation using standardized tools was not performed, as such instruments are not methodologically validated for descriptive, non-comparative studies. Accordingly, data were synthesized qualitatively, with the aim of providing an exploratory overview of diagnostic strategies, surgical approaches, and perioperative outcomes.

The protocol was registered in PROSPERO with ID number: CRD420251140106. As a review, formal approval from the institutional Ethics Committee was not required.

### 2.2. Data Extraction

After deduplication, the titles and abstracts were first screened independently by two reviewers to assess eligibility based on predefined inclusion and exclusion criteria. Discrepancies were resolved through discussion with a third reviewer. Articles deemed potentially relevant were included in the full-text screening phase, during which the complete manuscripts from all authors were retrieved and reviewed in depth. Using a standardized table, the following data were extracted: authors; years of publication; type of study; number of patients treated; diagnostic work-up; site and size of tumor; surgical details; perioperative management; intra and post-operative complications; and follow-up.

### 2.3. Statistical Analysis

Due to the data’s heterogeneity and retrospective nature, only descriptive statistics were performed: continuous variables were reported as mean ± standard deviation or median (range), and categorical variables as absolute numbers and percentages.

## 3. Results

### 3.1. Study Selection

**Figure 1:**
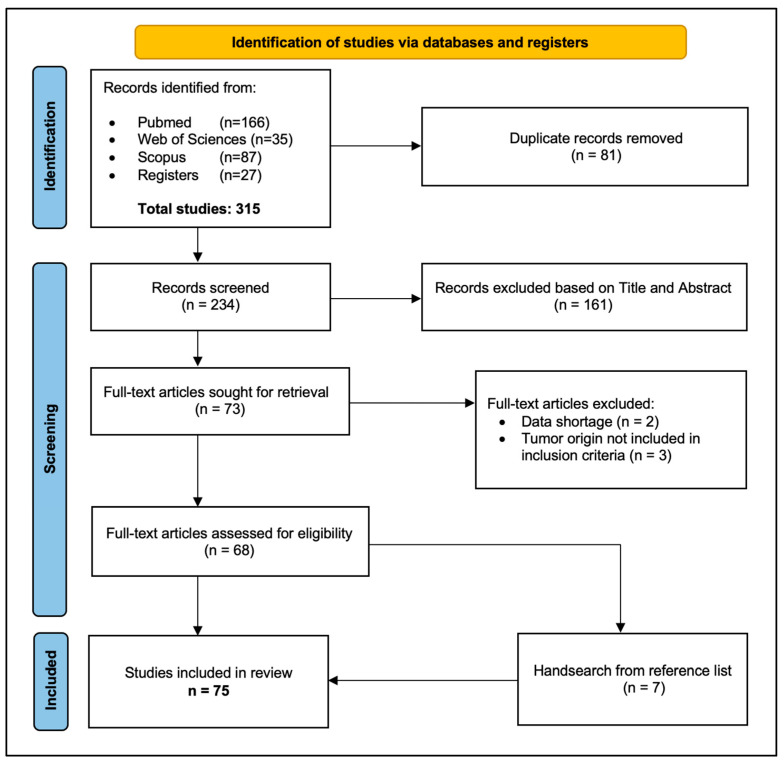
PRISMA 2020 flow diagram for the systematic review.

Search engines returned 315 potentially relevant articles, of which 234 were screened after removing 81 duplicates. After an eligibility assessment, another 166 articles were excluded, and 75 papers were ultimately included in this review. The PRISMA flow diagram of identification, screening, eligibility assessment, and inclusion of studies in the analysis is shown in Figure 1.

### 3.2. Synthesis Characteristics of Patients

Unless otherwise specified, all percentages reported in this review refer to the number of patients. Among 75 papers, a total of 79 patients were included in the analysis. Primary outcomes collected (main objectives) were: (I) The diagnostic work-up, (II) Preoperative management, (III) Surgical access, (IV) Perioperative complications and follow-up. Among 79 patients with a median age of 50 years (mean age: 49.3 ± 14.8 SD; range: 18–81 years) and a female predominance (62%), approximately half (50.6%) presented with aspecific symptoms. Most tumors were in the middle mediastinum (51.9%), followed by the posterior mediastinum (29.1%), anterior and superior mediastinum (10.1% and 11.4%, respectively), and neck (5.1%). Regarding diagnostic work-up, CT scan was used in 98% of patients, MRI in 40%, and FDG-PET in 41.8%, while echocardiography, angiography, and coronary angiography were selectively performed (16.5%, 19%, and 11.4%, respectively).

Sternotomy was the predominant surgical approach used (44.3%), with ascending aorta resection and re-anastomosis required in 7.6% of cases; cardiopulmonary bypass was utilized in 27.8% of overall cases. Thoracotomy was performed in 22.8%, while minimally invasive thoracic surgery approaches [video-assisted thoracoscopic surgery (VATS) and robotic-assisted thoracoscopic surgery (RATS)] were less frequently employed (19% and 6.3%, respectively).

Postoperative complications are described in 22 of 79 resected patients (27.8%). The most frequent complication was left vocal cord palsy (12.7%), and hemorrhage was reported in 3.8% of patients. The median follow-up was 12 months, ranging from 0 to 120 months. Table 1 summarizes the demographic, clinical, and surgical characteristics of the reported cases of MPs surgically treated.

## 4. Discussion

Surgery is the treatment of choice for resectable MPs [6,16], but surgical management is particularly complex due to the anatomical location and the potential secretion of catecholamines [8,17,18,19]. A surgical approach must be decided within a multidisciplinary team (thoracic surgeon, cardiac surgeon, cardiac anesthesiologist, intensivist, and endocrinologist), since the procedure can be complex with significant risk of life-threatening complications, such as intraoperative bleeding, due to its high vascularity and the tumor’s proximity to the major vessels and heart (atrium, vena cava, aorta, and pulmonary artery) [20,21,22,23,24,25]. Likewise, achieving a definitive preoperative diagnosis through biopsies is often challenging because of the same reasons. In Table 2 are listed the demographics, presentation, and management of MPs.

## 5. Preoperative Management

### 5.1. Clinical Presentation

Up to 50% or even 80% of patients with mediastinal paragangliomas are often asymptomatic, and the MPs are frequently detected incidentally during imaging studies conducted for other conditions [2,5,26,27,28]. The symptoms, when present, generally are caused by local mass effect and depend on the tumor’s size and anatomical location affecting surrounding structures; such symptoms include cough [26,29,30], chest pain [30,31,32], dyspnea [5,29,30,32], hoarseness [5,26], dysphagia, and facial swelling [5,26,32]. About 25–70% of patients with extra-adrenal paragangliomas may exhibit signs of excessive catecholamine secretion (functional tumors) [33,34,35] with hypertension [26,28,36,37], palpitations [26,28,36,38], headaches [26,27,36,38], profuse sweating or hyperhidrosis [26,28,36,37,38], tachycardia, anxiety, facial flushing, hyperglycemia [39], and nausea [26,33,37,38,40,41,42]. Even functional tumors can sometimes be asymptomatic [33,37]. Notably, reported proportions of functional versus non-functional paragangliomas vary widely across the literature referring to extra-adrenal paragangliomas rather than MPs. Available data suggest a predominance of non-functional tumors in MPs (about 80%), although the exact percentage remains uncertain due to the limited number of cases.

### 5.2. Imaging Presentation

**Figure 2:**
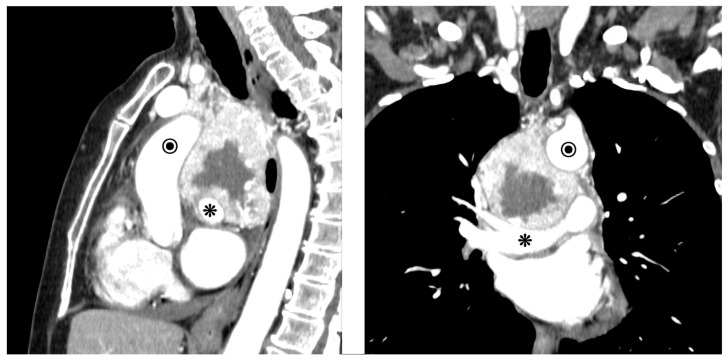
Coronal and sagittal computed tomography images showing a well-defined posterior mediastinal paraganglioma, surrounding pulmonary artery (asterisk) and ascending aorta (encircled) [Original images based on the authors’ personal surgical experience].

Due to their proximity to critical mediastinal structures and potential vascular involvement, careful radiological assessment is essential for preoperative planning and risk evaluation. Anatomical imaging by contrast-enhanced computed tomography of the thorax (thorax-CT scan), magnetic resonance imaging of the thorax (Thorax-MRI), functional imaging by positron emission tomography (PET), and single photon emission computed tomography (SPECT) are the mainstays of diagnosis of MPs [43,44]. The tumors usually appear well-demarcated and hyper-vascularized, with necrosis in the core, and are often located in the posterior mediastinum, near the sympathetic chain or great vessels (aorta, vena cava, pulmonary artery or vein). Both CT scan (the standard diagnostic process in all reported cases) and MRI play a significant role in the diagnosis of mediastinal paragangliomas, offering details about the tumor’s characteristics, location, and relationship with surrounding structures (Figure 2).

Compared to CT, MRI offers superior soft tissue contrast and characterization, making paragangliomas more discernible due to their distinct signal characteristics and the presence of flow voids in adjacent vessels [45]; this helps to distinguish paragangliomas from other hyper-vascular lesions [2]. The “salt and pepper” characteristic is well described in T2-weighted MRI, where high-signal areas reflect slow flow or hemorrhage (“salt”), and hypointense flow voids indicate fast-flowing intra-tumoral vessels (“pepper”) [2,38,46].

### 5.3. Metabolic Imaging

MPs display variable genetic alterations, resulting in distinct clusters with specific tumor locations, biochemical profiles, and cellular features. Because of this variability, appropriate imaging is required for diagnosis and follow-up of MPs. The tumors often overexpress somatostatin receptors (SSTR), making ^68^Ga-DOTA-SST PET highly effective for diagnosis. Other useful radionuclides include 18F-FDOPA, ^123^I-MIBG, and 18F-FDG. The ^68^Ga-DOTA-SST PET is the functional imaging modality of choice for most paragangliomas, across the spectrum of genetic mutations, while ^123^I-MIBG SPECT/CT remains the first choice when a theranostic approach is planned. The diagnostic performance of 18F-FDOPA PET can be influenced by tumor location and genetic status. Indeed, 18F-FDG PET is primarily recommended for metastatic lesions, particularly those associated with succinate dehydrogenase (SDHx) mutations or cases with an unknown or negative genetic profile [47,48,49,50]. A systematic review and meta-analysis corroborate this, reporting a pooled detection rate of 93% (95% CI: 91–95%) for ^68^Ga-DOTA-SSA PET/CT. This was significantly higher than the rates for 18F-FDOPA PET/CT (80%; 95% CI: 69–88%), 18F-FDG PET/CT (74%; 95% CI: 46–91%), and ^123^/^131^I-MIBG scintigraphy (38%; 95% CI: 20–59%) [50,51]. Regarding follow-up, nuclear imaging continues to play a critical role because it is minimally affected after surgery, enabling the accurate diagnosis of tumor recurrence or metastasis that could be missed by anatomical imaging alone [43].

### 5.4. Angiography and Coronary Angiography

Coronary angiography (CorA) is used in the preoperative assessment, playing an important role in identifying the tumor’s vascularization and its relationship to major blood vessels [45,52]. CorA reduces the risk of significant hemorrhage during surgical resection [53] by allowing the preoperative identification of the vessels feeding the tumor [54]. Many authors have used CorA, not as a diagnostic test to identify the tumor itself, but to plan the vessels’ cuts during the surgical procedure to reduce accidental damage [55]. Often a potential bloody supply originates from the coronary arteries [56,57]. Intraoperative use of an energy device or titanium clips improves the bleeding control, helping to maintain a clear surgical bed [55].

### 5.5. Preoperative Embolization

While there is no universally accepted formal evidence for MP embolization, the available literature suggests its utility in specific contexts, especially in highly vascularized and larger MPs [36,58]. Although preoperative embolization is described as a technique to reduce intra-operative bleeding by approximately 60–70% [7,30,59,60], there is a lack of consensus regarding this technique, and its application is less consistently demonstrated, suggesting an individualized approach [36]. If scheduled, it can be performed 1 to 7 days before surgery to achieve tumor cytoreduction [30,59].

Although generally safe, potential risks of neurological or vascular complications have been reported, such as pleuritic pain, device migration (which could lead to stroke in about 3% of cases), and spinal cord ischemia due to inadvertent embolization of spinal arteries [59,61]. Furthermore, Nam and coworkers highlight a non-negligible risk that tumor necrosis from embolization could induce an uncontrollable hypertensive crisis in functional tumors due to catecholamine release [36]. Experience in paragangliomas from other locations supports the benefit in bleeding reduction, but the decision should be personalized (evaluating case by case) and discussed in a multidisciplinary team [4,62,63,64,65].

### 5.6. Preoperative Biopsy

Most authors indicate that preoperative biopsy is often avoided due to significant risks of severe hemorrhagic complications and life-threatening hemodynamic instability (potential release of catecholamines during tumor manipulation) [2,7,27] that recur in about 10% of cases, while also proving non-diagnostic in a substantial proportion (up to 20%), thereby limiting their clinical utility [2]. The diagnosis of MPs relies on clinical assessment, biochemical testing (catecholamines and metanephrines in plasma or urine), and advanced imaging, which usually obviates invasive procedures, with definitive histopathological confirmation provided by surgical resection, which remains the treatment of choice [7,27,66]. Similarly, endobronchial ultrasound trans-bronchial aspiration (EBUS-TBNA) for biopsy of MPs must be approached with caution for the same reason. In this review analysis, the EBUS-TBNA was used in approximately 1/3 of cases, with 10% of complications, and was non-diagnostic in 20% of cases [2].

Although generally discouraged, biopsy may be considered in select cases, such as ambiguous imaging or recurrent tumors [5,67]; however, even in such situations, extreme caution is advised. In our review of 79 cases evaluated, 27 (34%) of them were biopsied by surgery in 12 cases (15%), CT-FNAB in 7 (9%), and EBUS-TBNA or EUS in 4 (5%) cases.

### 5.7. Perioperative Medical Management

Perioperative hemodynamic instability is also a significant concern during and after surgery for these tumors [68], requiring close collaboration between surgeons, anesthesiologists, and endocrinologists in cases of catecholamine-secreting tumors. The use of preoperative alpha-blockers is recommended to reduce the risk of cardiovascular complications [68,69].

## 6. Surgical Approaches

Surgical resection is the treatment of choice for patients with localized MPs since it is potentially curative [16]. The goal of surgery is to achieve oncological radicality (R0), as this remains the only curative option and is essential to minimize the risk of local recurrence and/or metastatic spread [5], even when extended resections are required in selected cases (infiltrative tumor).

However, the decision to proceed with surgery necessitates careful planning given the high risk of perioperative complications [4,7,12,13,62,70]. Preoperative multidisciplinary discussion plays a crucial role in planning the surgical approach and selecting the most appropriate surgical technique to manage potential complications. The surgical approach is chosen based on several factors, including the tumor’s size, location, vascularity, and its relationship to surrounding structures [71,72]. Both open and minimally invasive techniques are employed, although the thoracoscopic approach is more effective in small lesions, without involvement of surrounding structures. Massive intraoperative bleeding is the primary risk, and it may require extracorporeal circulation in more complex cases [1,5,7,55,62,73,74].

### 6.1. Cardiopulmonary Bypass

Cardiopulmonary bypass (CPB) has been reported as an effective tool in the surgical resection of MPs, and its use should be carefully evaluated considering tumor size, anatomical location, and the relationship with surrounding cardiovascular structures, such as the heart and great vessels [2,75]. Based on the descriptive analysis of the included cases, CPB was most frequently used in the presence of large tumors, posterior or central mediastinal location, and close anatomical relationship with major vessels, including the ascending aorta, pulmonary artery, or cardiac chambers. Given the absence of comparative studies, these observations should be interpreted as hypothesis-generating rather than evidence-based indications. However, the advantages of CPB include (I) facilitating resection, (II) managing hemorrhagic situations, (III) enabling vascular or cardiac reconstruction, and (IV) providing a cardiac arrest.

(I) CPB allows for better exposure and manipulation of the tumor, particularly when it adheres to or invades critical structures like the heart, aorta, pulmonary artery, or other great vessels, improving surgical site control and facilitating resection [10,55,76,77,78,79]. This enables surgeons to fully mobilize the vessels and achieve complete tumor removal [10,80].
(II) CPB provides a controlled environment to manage the risk of massive intraoperative bleeding, allowing for better blood loss control and providing circulatory support if extensive bleeding occurs [10].
(III) In cases of extensive resection or rupture of the mediastinal vessels due to forceful manipulation, CPB allows for hemorrhage control and their possible reconstruction with enhanced safety.
(IV) For tumors deeply involved with cardiac structures, CPB can facilitate temporary cardiac arrest, offering a motionless, bloodless field for precise dissection and resection [55,77]. In our series, the ascending aorta was transected in 6 (7.6%) cases and pulmonary artery in 4 (5.1%) patients. While preoperative embolization can significantly reduce the blood supply to the tumor and facilitate surgical resection, it does not always eliminate the need for CPB [67,78,81].

**Figure 3:**
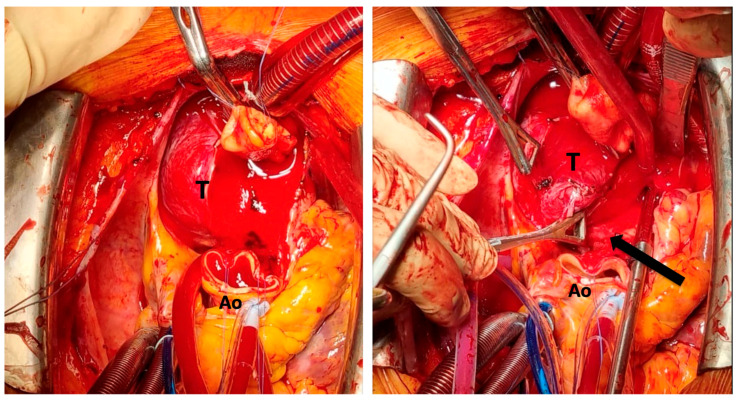
Cardiopulmonary bypass for the management of a posterior mediastinal paraganglioma (T) after transection of ascending aorta (Ao) to improve access and control of right pulmonary artery (arrow) [Original images based on the authors’ personal surgical experience].

Although some smaller or less complex tumors can be resected without CPB, its availability and planned use are critical for ensuring patient safety and achieving complete resection in challenging cases [67]. In the present review, CPB was utilized in 22 (27,8%) patients with predominantly posteriorly located big tumors (Figure 3).

These observations on CPB should be interpreted as hypothesis-generating rather than evidence-based recommendations, given the descriptive nature of the available data.

### 6.2. Median Sternotomy

Median sternotomy is a frequently used open surgical approach, particularly for large MPs or those located near major vessels like the heart, aorta, vena cava, and pulmonary artery [82]. It provides excellent exposure to these critical areas and may be performed with or without cardiopulmonary bypass, especially when complete resection of highly vascular lesions is required [71,78]. The sternotomy approach is strongly indicated in large tumors located posterior to the ascending aorta and surrounding the right branch of the pulmonary artery, where transection of the ascending aorta may be required, which is then re-anastomosed following tumor removal [22,55,83]. For this purpose, sternotomy offers a wide and safe surgical field. In our review, the authors used sternotomy in 35 (44%) patients out of all 79 cases.

### 6.3. Thoracotomy

Like sternotomy, a posterolateral thoracotomy is also commonly used, especially for posterior mediastinal tumors, allowing for better local control [84,85,86,87,88]. This approach is indicated when the tumor exhibits predominant extension into one hemithorax and can be fully managed from that side, including any deep or contralateral involvement [89]. In our review, the authors used a thoracotomy approach in 18 (23%) of the cases screened.

### 6.4. Video-Assisted Thoracoscopic Surgery

VATS offers benefits such as reduced trauma, less post-operative pain, faster recovery, and shorter hospitalization [86,90]. It has been successfully used for complete resection of MPs, particularly for smaller tumors or those that are not directly connected to vascular structures [91,92,93], even though it can be challenging for highly vascularized or larger tumors [66]. A VATS approach might be initially attempted but converted to thoracotomy if significant bleeding or complex adherence to structures is encountered [66,94]. In our review, the authors used a VATS approach in 15 (19%) of all cases studied.

### 6.5. Robotic-Assisted Thoracoscopic Surgery

RATS is a minimally invasive approach that utilizes robotic systems to enhance precision and visualization. Although the decision to perform RATS must be made with caution, a robotic procedure has been reported for the resection of MPs, including those in challenging locations like the subcarinal space [95,96,97], allowing for precise dissection and being advantageous in complex anatomical situations [32,98]. In our review, the authors used a RATS approach in 5 of all 79 patients (6.3%), although this technique will become more useful in the future.

Minimally invasive surgery (both VATS and RATS) has been used in selected cases, especially for small lesions and in favorable positions, with comparable results in terms of oncological radicality and a reduction in perioperative complications.

### 6.6. Other Approaches

Other approaches have been described, such as thoraco-abdominal and clamshell access [99,100]. Although rare, the choice of these approaches is determined by the location and size of tumors.

Through analysis of all cases reported in the literature over the last 20 years, we have collected and summarized the crucial steps, including diagnostic protocols, preoperative management, and the surgical plan, to propose a prudent pathway for the operative management of patients with MPs. Table 3 summarizes the main clinical claims regarding MPs and the corresponding strength of evidence.

## 7. Postoperative Outcomes and Complications

Operative mortality of surgical treatment of MPs is low, but morbidity can be significant in cases of large or secreting tumors. The morbidity and mortality rates after surgical removal of MPs can vary, reflecting the complex nature of these tumors, their high vascularity, and their proximity to vital structures.

### 7.1. Morbidity Rate

Surgical removal of MPs is associated with a notable rate of postoperative complications. Kanj and coworkers found that two-thirds of patients experienced adverse events from surgery [2]. One of the major postoperative complications is vocal cord paralysis, followed by arrhythmias, bleeding from the surgical site, hypertensive crisis, neurological deficits, and infections. Due to heterogeneous reporting and the absence of standardized complication grading across studies, perioperative complications were recorded as reported by the original authors, without formal distinction between minor and major events. In Table 4, we reported the most common perioperative complications found in this review of surgically treated patients.

### 7.2. Mortality Rate

Related mortality rate is around 16% during the follow-up period, while the intraoperative mortality range is 2–7% [1,2]. The most common cause of intraoperative mortality is uncontrollable bleeding from the tumor, such as from surrounding vessels or tumor bed. This not-negligible rate of intraoperative mortality justifies the use of cardiopulmonary bypass, which allows for greater control of both hemorrhage and vessel reconstruction, although these reported intraoperative mortality rates largely derive from historical series and highly selected complex cases.

## 8. Recurrences and Follow-Up

Although R0 surgery has a curative intent, allowing for low risk of local recurrence and distant metastasis, it is well established that MPs may recur even after apparently complete surgical excision, underscoring the need for strict and lifelong follow-up, as they have clinically relevant potential for both local recurrence and metastatic spread [101]. The average local recurrence for MPs has been estimated at 15% ± 7% at five years and 23% ± 9% at ten years following resection [78]. Other series describe local or metastatic relapse in 17% to 20% of cases [59]. In secretory paragangliomas, the five-year recurrence rate may approach 20% [62]. Additional studies report recurrence rates ranging from 3% to 16% after surgical removal [102], while a systematic review suggested an annual recurrence risk of approximately 1% [103,104]. These heterogeneous behaviors of paragangliomas reinforce the importance of prolonged follow-up in all affected patients. In Table 5 we show the recurrence-related risk factors after MP removal.

Genetic information, including SDHB mutation status, was inconsistently reported across the included studies and was unavailable in a substantial proportion of cases. As a result, no meaningful correlation between genetic background, surgical complexity, or recurrence risk could be explored in the present review, and genetic considerations remain largely extrapolated from the broader paraganglioma literature.

Surveillance typically involves both biochemical testing and imaging studies. It is strongly recommended to perform the annual monitoring of fractionated metanephrines and catecholamines in plasma or 24-h urine for life [1,105,106], as well as 123I-MIBG and FDG-PET scans [43,107,108,109].

The relatively short median follow-up observed in this review (12 months; range 0–120 months) reflects the characteristics of the data provided by literature, in which long-term surveillance is inconsistently reported, particularly in case reports (Table 4). Consequently, recurrence rates derived from the present analysis should be interpreted with caution, as late recurrences may be underreported. This limitation further supports the recommendation for lifelong follow-up (clinical, biochemical, and imaging) in all patients with MP, even after apparently complete surgical resection.

### Follow-Up Frequency Guidelines

Some guidelines suggest follow-up every three months for the first year, biannually for three years, and then annually for 10 years [95]. The European Society of Endocrinology recommends a ten-year follow-up with annual laboratory testing [9].

## 9. Study Limitations

The evidence derived from the present review should be interpreted with caution, given the intrinsic limitations of the available literature.

(I) The data from most of the literature consists of case reports or small retrospective series, often lacking standardized inclusion criteria, comparative groups, or prospective data collection. Nevertheless, the overall methodological quality of the available studies is generally acceptable, and retrospective series and literature reviews generally report largely consistent findings.
(II) Due to the rarity of thoracic and mediastinal paragangliomas, the literature remains limited to single-center experiences, which does not allow the definition of formal levels of evidence. Even so, in the context of rare diseases, the cumulative evaluation of complex and isolated cases managed over long periods can offer relevant clinical information, particularly regarding surgical feasibility and perioperative management. This is exemplified by the present twenty-year single-center experience.
(III) Management strategies described in the literature—most notably surgical resection via median sternotomy—reflect expert opinion and historical practice. In addition, heterogeneity in reporting, limited sample sizes, and incomplete follow-up further restrict the strength of any definitive clinical conclusions.
(IV) Follow-up duration was highly varied and frequently limited, reflecting the retrospective and case-based nature of the available literature. This limits the ability to draw definitive conclusions regarding long-term recurrence and survival outcomes.
(V) Finally, future multicentric registries and prospective observational studies are needed to consolidate the existing evidence and to support the development of more standardized treatment recommendations for thoracic and mediastinal paragangliomas.

However, no meta-analysis was feasible due to the heterogeneity and descriptive nature of the available data; therefore, the conclusions of this review should not be interpreted as guideline-level recommendations.

## 10. Conclusions

In conclusion, surgical treatment with R0 resection remains the cornerstone of treatment for MPs, associated with favorable long-term local disease control and low recurrence rate, despite significant surgical challenges related to complex anatomy, vascularity, and potential hemodynamic instability. However, the evidence summarized in this review is derived predominantly from retrospective case reports and small case series. Therefore, the findings should be interpreted with appropriate caution and considered primarily descriptive and hypothesis-generating.

The optimal surgical approach, including the potential role of cardiopulmonary bypass and minimally invasive techniques, should be individualized within a multidisciplinary setting to minimize perioperative complications. Future multicenter registries and prospective observational studies are warranted to better define standardized management strategies.

## Figures and Tables

**Table 1:** Characteristics and diagnostic work-up of 79 patients with mediastinal paraganglioma.

Variable	Value
Total number of cases, n.	79
Mean age ± SD (years; range)	49.3 ± 14.8 (18–81)
Median age (years)	50.0
Female, n. (%)	49 (62)
Clinical presentation	n. (%)
Hypertension-related symptoms	32 (40.5)
Palpitations	13 (16.5)
Headache	11 (13.9)
Aspecific symptoms	40 (50.6)
Mediastinum Localization	n. (%)
Medium	41 (51.9)
Posterior	23 (29.1)
Anterior	9 (11.4))
Superior	8 (10.1)
Neck involvement	4 (5.1)
Diagnostic work-up	n. (%)
Thorax CT-scan	78 (98.7)
MRI	32 (40.5)
FDG-PET	33 (41.8)
Angiography	13 (16.5)
Echocardiogram	15 (19)
Coronarography	9 (11.4)
Preoperative biopsy	30 (38)
Biopsy Details	n. (%)
Surgical biopsy	12 (15.2)
CT-FNAB	7 (8.9)
EBUS/EUS	4 (5.1)
Ultrasound-FNAB	4 (5.1)

**Table 2:** General demographics characteristics, presentation, and management of mediastinal paraganglioma.

Feature	Description
Typical age at diagnosis	30–60 years (wide variation possible)
Gender	Female predominance
Location	Posterior mediastinum (most common), anterior and middle mediastinum, intrapericardial (rare)
Functional vs. Non-Functional	~80% of MPs are non-functional and asymptomatic
Symptoms mass-related	Compressive: cough, dyspnea, dysphagia, chest pain
Functional symptoms	Palpitations, hypertension, sweating
Associate genetic Syndrome	MEN2, VHL, SDHB/SDHD mutations, NF1
Imaging—CT scan	Well-circumscribed, hyper-vascular mediastinal mass with contrast enhancement; central necrosis.
Imaging—MRI scan	The same characteristics of CT (see above)
Imaging—PET (FDG o 68Ga-DOTATATE)	High uptake; useful for detecting multiple or metastatic lesions, and follow-up
Angiography (if indicated)	High vascularization and blood supply
Biopsy	Generally contraindicated due to bleeding risk
Cardiopulmonary bypass	Generally indicated (posterior site and big size, with vascular involvement)
Treatment	Complete surgical resection (R0)
Prognosis	Good if R0 achieved; higher recurrence/metastasis risk

**Table 3:** Summary of evidence on the management of mediastinal paragangliomas.

Statement	Evidence Strength	Rationale
Surgical resection with R0 is the gold standard for MPs.	Strong	Supported by NCCN guidelines, large case series with low recurrence rates.
Sternotomy and thoracotomy allow for greater exposure and control of MPs.	Moderate	Several studies suggest this approach for posterior lesions with several involved vascular structures.
VATS and RATS are safe in selected cases.	Moderate	Recent studies show comparable outcomes to traditional surgery for small/localized tumors.
Cardiovascular complications are frequent in secreting tumors.	Moderate	Multicenter reviews and clinical cases describe intraoperative hemodynamic instability.
Cardiopulmonary bypass can reduce the intraoperative hemorrhage risk.	Weak	Most evidence derives from case reports and case series.
Long-term follow-up is mandatory, especially in high-risk patients.	Strong	Recommendations from scientific societies and ENS@T registry data.
Operative mortality is low, but morbidity can be significant.	Moderate	Case series and systematic reviews.
High-quality prospective randomized studies are lacking.	Weak	Most evidence derives from retrospective studies and case reports.

R0: oncological radicality; MPs: mediastinal paragangliomas; NCCN: National Comprehensive Cancer Network; VATS: video-thoracoscopic surgery; RATS: robotic-assisted thoracic surgery; ENS@T: European Network for the Study of Adrenal Tumours.

**Table 4:** Surgical management and postoperative outcomes of 79 patients with mediastinal paraganglioma.

Variable	Value
Surgical approach	n. (%)
Sternotomy	35 (44.3)
Thoracotomy	18 (22.8)
VATS	15 (19)
RATS	5 (6.3)
Aorta section	6 (7.6)
Pulmonary artery section	4 (5.1)
Cardiopulmonary bypass, n. (%)	22 (27.8)
Tumor Characteristics	
Mean tumor size ± SD (mm)	57.6 ± 25.3
Range (mm)	5–131
Postoperative complications, n. (%)	22 (27.8)
Left vocal cord paralysis	10 (12.7)
Cardiovascular adverse events	4 (5.1)
Hemorrhage	3 (3.8)
Respiratory failure	3 (3.8)
Wound infection or dehiscence	2 (2.5)
Others	3 (3.8)
Follow-up	
Median follow-up, months (range)	12 (0–120)
Case with follow-up ≥ 12 months, n. (%)	28 (35.4)

**Table 5:** Clinical and Biological Predictors of Recurrence in Paraganglioma.

Risk Factors	Implications
Familiar Paraganglioma	Increased recurrence rate compared to sporadic cases
Tumor Size > 5cm	Related to risk factor for recurrence
Younger Age	Is an independent risk factor for recurrence
SDHB Genetic Mutations	Significantly increases the risk of recurrences and metastasis
Extra-Adrenal Location	Negative prognostic factor

SDHB: succinate dehydrogenase subunit B.

## Data Availability

No new data were generated or analyzed in this study. Data sharing is not applicable to this article.

## Supplementary Materials

The following supporting information can be downloaded at: https://www.mdpi.com/article/10.3390/cancers18030486/s1, Full search strategies and exact search strings, S1.

## Abbreviations

The following abbreviations are used in this manuscript:

MPs	Mediastinal Paraganglioma
SD	Standard Deviation
CT scan	Computed Tomography
MRI	Magnetic Resonance Imaging
FDG-PET	Fluorodeoxyglucose—Positron Emission Tomography
VATS	Video-assisted Thoracoscopic Surgery
RATS	Robotic-assisted Thoracoscopic Surgery
CT-FNAB	Computed Tomography—Fine Needle Aspiration Biopsy
EBUS	Endobronchial Ultrasound
EUS	Esophageal Ultrasound
MEN2	Type 2 Multiple Endocrine Neoplasia
VHL	Von Hippel-Lindau
SDHB/C	Succinate Dehydrogenase B or C
NF1	Neurofibromatosis di type 1
SPECT	Single Photon Emission Computed Tomography
CorA	Coronary angiography
CPB	Cardio-pulmonary bypass
