# Supplementary material for: Surgical Approaches and Perioperative Outcomes in Mediastinal Paragangliomas: A 20-Year Comprehensive Systematic Review
Source: Cancers (Basel). 2026 Feb 1;18(3):486. doi: 10.3390/cancers18030486 (PMC12896465; doi:10.3390/cancers18030486)
Supplement: Supplementary file 1 [file cancers-18-00486-s001.zip › cancers-4098931-supplementary.pdf]

Supplementary File S1. Full search strategies and exact search strings

The literature search was conducted using PubMed, Web of Science, and Scopus.

Searches were limited to studies published between January 2005 and December 2025.

Filter years from 2005 to 2025

The search plan was detailed in the methods section of the manuscript.

PUB MED
Search strategy (2005–2025):

("Paraganglioma"[MeSH] OR "Extra-adrenal paraganglioma" OR "Thoracic paraganglioma" OR "Mediastinal paraganglioma")
AND
("Thorax" OR "Mediastinum")
AND
("Surgery"[MeSH] OR "Surgical resection" OR "Surgical treatment")

Web of Sciences
Search strategy (2005–2025):

TS=("Paraganglioma" OR "Extra-adrenal paraganglioma" OR "Thoracic paraganglioma" OR "Mediastinal paraganglioma")
AND
TS=("Thorax" OR "Mediastinum")
AND
TS=("Surgery" OR "Surgical resection" OR "Surgical treatment")
AND
PY=(2005-2025)
AND
WC=("Surgery")
NOT
DT=("Meeting Abstract")

Scopus
Search strategy (2005–2025):

TITLE-ABS-KEY("paraganglioma" OR "extra-adrenal paraganglioma" OR "thoracic paraganglioma" OR "mediastinal paraganglioma")
AND
TITLE-ABS-KEY("thorax" OR "mediastinum")
AND
TITLE-ABS-KEY("surgery" OR "surgical resection" OR "surgical treatment")
AND
PUBYEAR > 2004 AND PUBYEAR < 2026
AND
(SUBJAREA("MEDI") OR SUBJAREA("SURG"))
